# Severe Toxic Effects on Pelagic Copepods from Maritime Exhaust Gas Scrubber Effluents

**DOI:** 10.1021/acs.est.0c07805

**Published:** 2021-04-20

**Authors:** Peter Thor, Maria E. Granberg, Hulda Winnes, Kerstin Magnusson

**Affiliations:** † Fram Centre, Norwegian Polar Institute, 9296 Tromsø, Norway; ‡ IVL Swedish Environmental Research Institute, Kristineberg Marine Research Station, Kristineberg 566, 451 78 Fiskebäckskil, Sweden; ¶ IVL Swedish Environmental Research Institute, Aschebergsgatan 44, 411 33 Gothenburg, Sweden

## Abstract

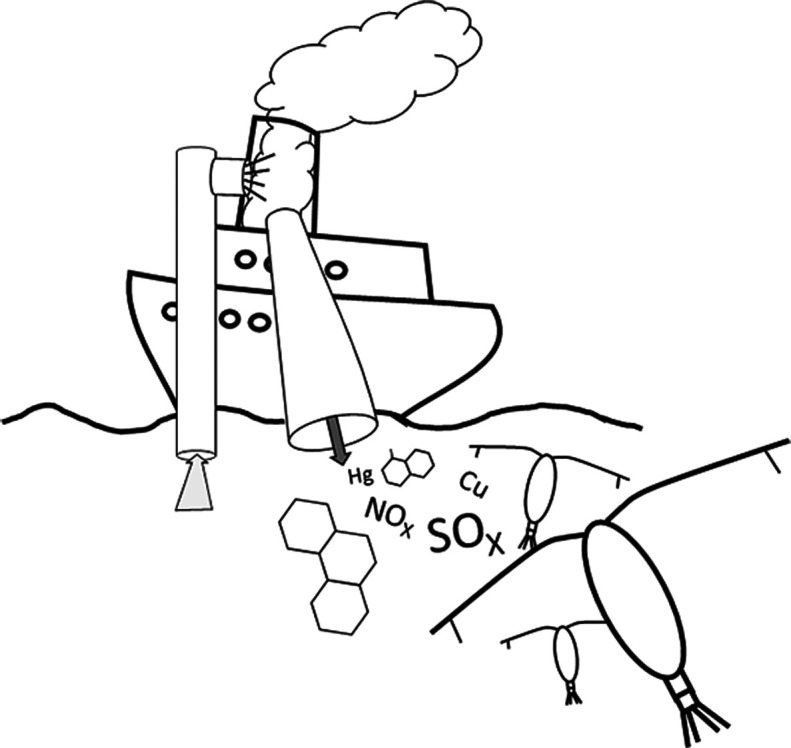

To reduce sulfur emission from global shipping, exhaust gas cleaning systems are increasingly being installed on board commercial ships. These so-called scrubbers extract SO_*X*_ by spraying water into the exhaust gas. An effluent is created which is either released directly to the sea (open-loop system) or treated to remove harmful substances before release (closed-loop system). We found severe toxic effects in the ubiquitous planktonic copepod *Calanus helgolandicus* of exposure to effluents from two closed-loop systems and one open-loop system on North Sea ships. The effluents contained high concentrations of heavy metals and polycyclic aromatic hydrocarbons (PAHs), including alkylated PAHs. We observed significantly elevated mortality rates and impaired molting already in the lowest tested concentrations of each effluent: 0.04 and 0.1% closed-loop effluents and 1% open-loop effluent. These concentrations correspond to total hydrocarbon concentrations of 2.8, 2.0, and 3.8 μg L^–1^, respectively, and compared to previous studies on oil toxicity in copepods, scrubber effluents appear more toxic than, for example, crude oil. None of the individual PAHs or heavy metals analyzed in the effluents occurred in concentrations which could explain the high toxicity. The effluents showed unexpected alkylated PAH profiles, and we hypothesize that scrubbers act as witch’s cauldrons where undesired toxic compounds form so that the high toxicity stems from compounds we know very little about.

## Introduction

Atmospheric emissions from ships in international traffic are often high compared to emissions from other transport modes. Less stringent regulations on sulfur content and lesser use of exhaust after-treatment on ships result in higher emissions of SO_*X*_, NO_*X*_, and particles from combustion of marine fuels.^1^ Concern has been raised that these emissions may pose serious threats to human and environmental health,^2−5^ and to accommodate these concerns, the International Maritime Organization has agreed on regulations to limit the sulfur content in marine fuels to 0.5% globally and 0.1% in Sulfur Emission Control Areas. However, regulations allow the use of high-sulfur heavy fuel oils if an exhaust gas cleaning system (a “scrubber”) is used to reduce atmospheric emissions of SO_2_ to equivalent levels.^6^ This last option is often more cost efficient in large ships,^7^ and installation of scrubbers has increased dramatically with an estimated 4500 ships equipped with one or more scrubbers by 2021.^8^ However, heavy fuel oils contain high concentrations of PAHs and heavy metals compared to distillates such as diesel, and their use may introduce excessive unwanted environmental consequences when the effluents from the scrubbers are released in the environment.

Scrubbers extract SO_*X*_ by spraying water into the exhaust gases before emission.^7,9^ The effluent created (exhaust gas scrubber effluent, EGSE, also called “wash water”) is most frequently released to the sea untreated or partially treated from the so-called open-loop systems, or it is treated in closed-loop systems to remove harmful substances and adjusted for acidity before discharge. Besides SO_*X*_, a wide range of other harmful substances, such as toxic hydrocarbons and heavy metals, are removed from the exhaust gas and instead released directly to the sea.^9,10^

Release of EGSE poses a significant potential risk to the marine environment.^11^ Nearshore waters are the most heavily trafficked, and these also hold the largest biodiversity.^12^ Fish and planktonic invertebrates living near the sea surface will experience the largest impact, but most marine animals spend their larval life in the water column and hence run the risk of EGSE exposure during this critical part of their life cycle. Many fish species spawn in the nearshore environment. This is also where they spend their larval and juvenile life, a period in which they are most vulnerable to negative effects from pollution.^13^ Despite this obvious risk, knowledge on the toxicity of EGSE and the effects of its release is, at present, virtually absent.^14−16^

In the present study, we analyzed medium-term effects on juvenile life stages of the calanoid copepod *Calanus helgolandicus* of exposure to EGSE from two closed-loop systems and one open-loop scrubber system. Calanoid copepods constitute up to 80% of the animal planktonic biomass worldwide and form a pivotal node in the pelagic food web. By their grazing on microplankton, they are the main conveyors of energy from lower trophic levels to the upper marine food web, and they form the bulk of the diet of many larval and juvenile fish, thereby supporting global stocks of many fish species.^17−21^ Any effects on copepod populations will therefore extend well beyond the copepods themselves.^22^

## Methods

### Incubations

EGSE was collected in acetone-washed (analysis grade, Sigma-Aldrich) 5 L glass bottles from the outlet from scrubbers on board three ships in service in the North Sea: in September 2017, from a closed-loop system (EGSE/CL1), and in May 2018, from one closed-loop system (EGSE/CL2) and one open-loop system (EGSE/OL). EGSE from the closed-loop systems consists of bleed-off and decanter water from the recirculating system, whereas EGSE from the open-loop system consists of the wash water from the scrubber. All three were collected at normal vessel cruising speed (70–75% engine load). Samples were stored cold (8 °C) and in the dark until experiments (within a week). Chemical analysis of the EGSEs is described in the Supporting Information (SI1).

*Calanus helgolandicus* were caught in the Gullmarsfjord, Swedish west coast (58° 16′ 44″ N, 11° 29′ 41″ E), using a 450 μm net equipped with a closed cod end and transported to the Kristineberg Marine Research Station (KMRS) within an hour. Juvenile stage CIII and stage CV copepodites were collected under the stereoscope using the number of abdominal segments and pleopods for stage identification. *C. helgolandicus* was distinguished from the partially sympatric *Calanus finmarchicus* by the curvature of the interior margin of the basipods of the fifth pleopod pair.

At KMRS, copepods were exposed to concentrations ranging from 0 to 5% for EGSE/CL1 and EGSE/CL2 and 0 to 40% for EGSE/OL. For each treatment, a 3 L batch of treatment water was prepared in a 5 L Erlenmeyer glass flask by mixing EGSE with 0.3 μm-filtered seawater collected at 40 m. For food, paste of the diatom *Thalassiosira weissflogii* (Reed Mariculture, Campbell, California, USA) was added to a final concentration of *ca.* 10 μg chlorophyll a (Chl *a*) L^–1^ to every batch. The chlorophyll concentration of the diatom paste was measured according to Strickland and Parsons.^23^ Before use, the treatment water batches were preinoculated for 24 h on gentle mixing to allow equilibrium between dissolved EGSE and EGSE adsorbed to algal cells.

At the onset of the incubation period, four replicate 620 mL glass bottles were filled from each treatment batch and eight stage CIII copepodites or five stage CV copepodites were added by pipetting. All bottles were then incubated on a rotating plankton wheel (0.5 rpm) at 8 °C in the dark for a total of 7 days for stage CV copepodites exposed to EGSE/CL1, 8 days for stage CIII copepodites exposed to EGSE/CL2, and 14 days for stage CIII copepodites exposed to open-loop EGSE/OL (Table S1).

Every day or every other day (Table S1), approximately 500 mL of water was reverse-filtered from each bottle by inserting a tube fitted with a 200 μm screen at the bottom into the bottle and siphoning off water through a piece of silicone tubing inserted into this tube. All copepods remained in the bottle and were subsequently poured into a Petri dish. Here, the number of live, dead, and lethargic individuals and the number of shed cuticles from molting were counted (except on day 3 through 5 for stage CV copepodites exposed to EGSE/CL1), and the copepod stage was determined under the stereoscope. Copepods turn from transparent to opaque within hours of their death, and transparent nonmoving individuals were classified as lethargic accordingly. New treatment water prepared the day before (as before the onset of the incubation period) was then filled into the bottles, the copepods were poured back into the bottle, and the bottle was replaced on the plankton wheel. For the last incubation day (second to last for the EGSE/OL test), additional control bottles without copepods were prepared with water from each treatment batch for estimates of ingestion rates.

For every water renewal, total scale pH (pH_T_), total alkalinity (A_T_), and temperature were measured in all bottles prior to the addition of copepods. pH_T_ was established from the electric potential (mV) of an HI 98183 pH/ORP meter (Hanna, Woonsocket, Rhode Island, USA) by a standard curve previously established for this electrode at similar temperature and salinity.^24^ A_T_ was measured by potentiometric titration of 25 mL samples in a Titroline potentiometric titrator (SI Analytics, Weilheim, Germany).^25^

### Ingestion Rate and Metabolic Rate

Metabolic rates were measured on the day before the last incubation day. Metabolic rates were estimated from the depletion of O_2_ in 1.6 mL vials fitted with fluorescent O_2_ optodes (PSt3 spots, PreSens, Regensburg, Germany) holding single copepods compared to O_2_ depletion in control vials with no copepods (four replicates for each treatment). Weight-specific metabolic rates were calculated according to Thor *et al.*(26) Also, on the day before the last incubation day (two days before in the EGSE/OL test), 100 mL samples were collected from newly prepared treatment water for algal cell concentration measurements for the ingestion rate measurements. Four extra bottles containing no copepods were prepared for ingestion rate controls. All bottles (minus the copepodites used for metabolic rate measurements) were then replaced on the plankton wheel. The number of individuals in the bottles varied between one and four depending on the mortality during the incubation period. Finally, at the end of the last day, the content of each bottle was poured into a 63 μm sieve to retrieve copepods, and the treatment water was collected from under the sieve for ingestion rate measurements. Algal cell concentrations were then measured using an electronic particle counter (Coulter Z3). Weight-specific ingestion rates were calculated according to Frost^27^ using a cell carbon mass of 64 pgC cell^–1^ for *T. weissflogii*.^28^ Finally, all copepods (including copepods used for metabolic rate measurements) were collected for stage determination and length measurement using a calibrated scale in the eyepiece of the stereoscope. Body masses were calculated using a *W* (μgC) = 1.95 × 10^–9^*L* (μm)^3.154^ weight/length relationship^29^ to facilitate calculation of weight-specific rates.

### Calculations and Statistical Analysis

Daily mortality was calculated as the fraction of dead copepods relative to the number of total copepods (live + lethargic + dead) on each day, and lethargy (stage CIII copepodites only) was calculated as the fraction of lethargic copepods relative to the number of total live copepods (live + lethargic) each day. Stage development was followed only in stage CIII copepodites since in *Calanus*, the CV stage is very much prolonged and variable among individuals.^30^ Development (molting) from stage CIII to CIV was estimated from the appearance of shed cuticles and live stage CIV copepodites in the bottles. Mortality rates (d^–1^) were calculated from linear regressions of the cumulative mortality from the first sampled day until and including the sampling day when the cumulative mortality reached its maximum. Later days were excluded to avoid erroneous underestimation. Molting rates (d^–1^) were calculated similarly. For every sampling day, LC_50_ (EGSE concentration) values were calculated as the half saturation constant (*K*_m_) from regressions on cumulative mortality *versus* EGSE concentration using the Hill sigmoid function, *m* = [EGSE]^h^/(*K*_m_^h^ + [EGSE]^h^).

For each of the three EGSEs, differences in cumulative mortality were tested among EGSE concentrations and days by 2-factor PERMANOVA on similarity matrices assembled using Euclidian distances with estimates of P using Monte Carlo tests for small sample sizes (*P*_MC_) in Primer 6+.^31^ Differences in mortality rates among EGSE concentrations or copepodite stage were examined by comparing cumulative mortalities using 1-factor PERMANOVA with day as the covariate.^31^ In these tests, significant interactions between the concentration or stage and day indicate significantly different rates.

Differences in LC_50_ among days were tested by 1-factor ANOVA on mean, sample size, and standard errors of *K*_m_ from the regressions (SigmaPlot 11.0).

Differences among EGSE concentration treatments in the ingestion rate and metabolic rate were tested by 1-factor PERMANOVA (Euclidian distance matrices) with estimates of *P* using Monte Carlo tests for small sample sizes (*P*_MC_).

All PERMANOVA tests were preceded by PERMDISP tests to verify homogeneity of dispersions and followed by pairwise comparisons among EGSE concentrations and days. All test results were judged significant using a significance level of 0.05.

## Results and Discussion

### Mortality

Both closed-loop and open-loop EGSEs were highly toxic to *C. helgolandicus* copepodites. While there was no mortality in any of the control treatments, all copepods died within 1 day when exposed to the 5% concentration of EGSE/CL1 and EGSE/CL2 and within 8 days when exposed to the 40% concentration of EGSE/OL (Figure S1).

**Figure 1:**
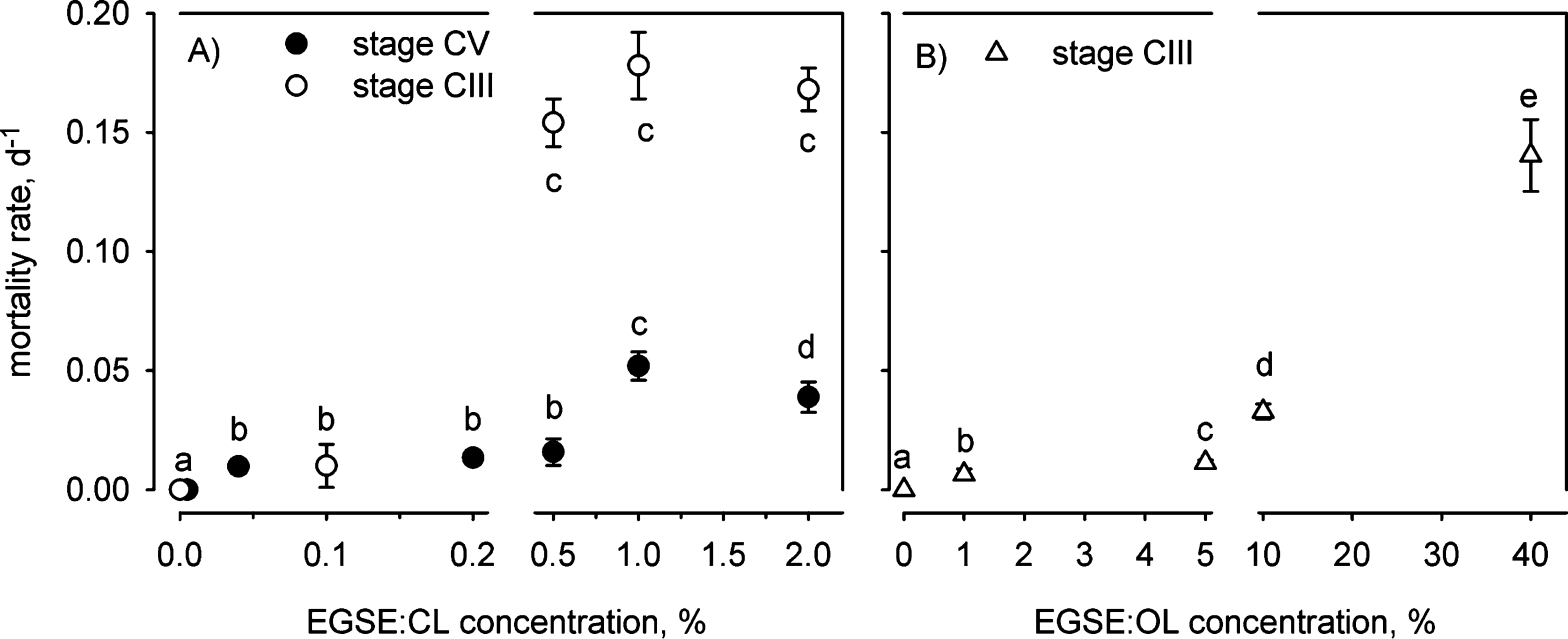
Mortality rates (means ± standard errors) of *Calanus helgolandicus* stage CIII and CV copepodites exposed to EGSE. Lowercase italicized letters indicate statistically equal groups. The 5% EGSE/CL treatments are not shown as all copepods died before the first sampling (Figure S1).

Mortality rates differed significantly among concentrations of all three EGSEs (1-factor PERMANOVA with day as the covariate: stage CV EGSE/CL1: Pseudo-*F*_5,170_ = 5.53, *P* < 0.0001; stage CIII EGSE/CL2: Pseudo-*F*_5,167_ = 31.2, *P* < 0.0001; stage CIII EGSE/OL: Pseudo-*F*_4,111_ = 19.7, *P* < 0.0001; Figure 1). We found mortality rates significantly different from the control already at the lowest tested concentrations of all three EGSEs: for stage CV copepodites at 0.04% EGSE/CL1 and for stage CIII copepodites at 0.1% EGSE/CL2 and 1% EGSE/OL (Figure 1). These concentrations corresponded to total hydrocarbon concentrations in the exposure water of 2.8, 2.0, and 3.8 μg L^–1^, respectively. In comparison, crude oil has shown no mortality in *C. finmarchicus* at total hydrocarbon concentrations up to ∼150 μg L^–1^.^32^ It seems that exposure during a period of several days to even very low concentrations of EGSE will have detrimental effects on copepod populations. Accordingly, while the standard 24 and 48 h LC_50_ values were *ca.* 2.6% in stage CIII copepodites exposed to EGSE/CL2, LC_50_ decreased significantly to as low as 0.045% on day 5 (1-factor ANOVA on *K*_m_ values: *F*_6,125_ = 37.5, *P* < 0.001; Table 1). Thus, standard LC_50_ may not be sufficient to evaluate effects of medium-term EGSE exposure. The lowest and earliest significant effect appeared at 0.1% on day 4 (lowest effect concentration at day 4, LOEC_4d_) (2-factor PERMANOVA pairwise tests: *t*_6_ = 4.09, *P*_MC_ = 0.0081). However, in stage CIII copepodites exposed to EGSE/OL, there were no differences in LC_50_ among days (1-factor ANOVA on *K*_m_ values: *P* > 0.05). For these, the lowest and earliest significant effect appeared at 5% on day 8 (LOEC_8d_) (PERMANOVA pairwise tests between day 2 and day 8: *t*_4_ = 11.5, *P*_MC_ = 0.0005), while LC_50_ was as high as 13.35% for that day (Table 1). For stage CV copepodites exposed to EGSE/CL1, there were no differences in LC_50_ among days (1-factor ANOVA on *K*_m_ values: *P* > 0.05) due to the regression model returning very high standard errors at the low concentrations. The lowest and earliest significant effect appeared at 2% at day 6 (LOEC_6d_) (PERMANOVA pairwise test between day 1 and day 6: *t*_6_ = 2.98, *P*_MC_ = 0.025). LC_50_ was 1.85% at day 6.

**Table 1:** LC_50_ Values (Means ± Standard Errors) of *Calanus helgolandicus* Stage CIII and CV Copepodites Exposed to EGSE^a^

	stage CV, EGSE/CL1			stage CIII, EGSE/CL2				stage CIII, EGSE/OL		
day	*n*	LC_50_ % concentration		*n*	LC_50_ % concentration		day	*n*	LC_50_ % concentration	
1	25.0	3.16 ± 517		31.5	2.57 ± 0.39	*a*	2	30.0	>40	
2	18.8	3.15 ± 1 467		28.3	2.59 ± 0.25	*a*	4	27.5	>40	
3				22.5	0.964 ± 0.180	*c*	6	23.8	15.01 ± 1.39	*a*
4				19.3	0.117 ± 0.093	*d*	8	21.5	13.35 ± 0.90	*a*
5				16.0	0.045 ± 0.027	*d*	10	21.5	12.24 ± 0.95	*a*
6	20.8	1.85 ± 0.30	*a*	15.0	0.072 ± 0.045	*d*	11	21.0	12.25 ± 1.37	*a*
7	14.0	1.73 ± 0.31	*a*				14	19.5	10.52 ± 1.38	*a*
8				13.0	0.075 ± 0.055	*d*				

a Values of *n* indicate the average number of individuals included in the four replicate LC_50_ regressions for each day. Lowercase italicized letters indicate statistically equal groups. EGSE/CL1 is the first closed-loop scrubber effluent, EGSE/CL2 is the second closed-loop effluent, and EGSE/OL is the open-loop effluent.

Only one previous study on the toxicity of EGSE to marine planktonic organisms exists. Koski *et al.*(16) tested acute 24 h effects of an open-loop scrubber system on lab cultures of the copepod *Acartia tonsa* and the cryptophyte *Rhodomonas*sp. and found increased mortality in adult female *A. tonsa* at EGSE concentrations of ≥10%. In comparison, we found only a slight increase in mortality after 48 h in the highest EGSE/OL concentration (40%). The discrepancy can be explained either by differences in sensitivity between the two species—*A. tonsa* is much smaller than *C. helgolandicus* with a much larger body surface over which toxic compounds can be absorbed relative to the body volume—or perhaps more likely by differences in the chemical composition of the EGSEs from the two studies.

### Stage Development

**Figure 2:**
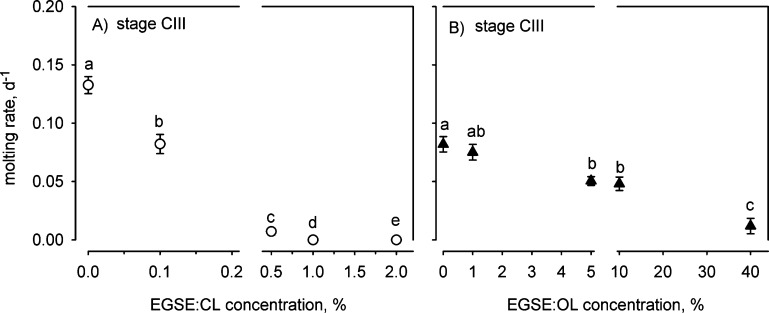
Molting rates (means ± standard errors) of *Calanus helgolandicus* stage CIII copepodites exposed to EGSE. Lowercase italicized letters indicate statistically equal groups. The 5% EGSE/CL treatment is not shown as all copepods died before the first sampling (Figure S1).

The increased mortalities were accompanied by significantly reduced molting of surviving stage CIII copepodites beginning at 0.1% EGSE/CL2 and 5% EGSE/OL (Figure S2). We found no shed cuticles in the three high EGSE/CL2 concentrations (1, 2, and 5%), only one single cuticle at the very first sampling in the 0.5% EGSE/CL2 concentration, and no subsequent molting (Figure S2A). Similarly, we found only one shed cuticle in the high EGSE/OL concentration (40%) at the very first sampling and no subsequent molting (Figure S2B). We also observed several copepodites showing signs of abnormal molting with remains of old cuticle on the antennules and two copepodites in the 5 and 10% EGSE/OL showing malformed antennules, and one might speculate that the increased mortality was induced by failure to molt properly. Molting rates decreased significantly from low to high concentrations of both EGSE/CL2 and EGSE/OL (1-factor PERMANOVA: EGSE/CL2: Pseudo-*F*_5,167_ = 30.4, *P* < 0.0001, EGSE/OL: Pseudo-*F*_4,111_ = 44.4, *P* < 0.0001; Figure 2) and were significantly reduced already at 0.1% in EGSE/CL2 and 5% for EGSE/OL (Figure 2).

To molt, the physiology of stage CIII copepodites is directed toward complex processes leading to the production of a new functioning exoskeleton. Our observations could indicate a malfunction of the production of the new cuticle in the later part of the molting cycle. In stage CV copepodites, molting to the adult stage takes place in spring after the winter diapause. We tested stage CV copepodites during autumn and cannot predict if any effects on molting exist also in this developmental stage. However, significantly lower mortality rates in stage CV copepodites exposed to EGSE/CL1 than in stage CIII copepodites exposed to EGSE/CL2 (2-factor PERMANOVA comparing the three similar closed-loop EGSE concentrations in the incubations of stage CIII and stage CV copepodites, 0.5, 1, and 2%: Pseudo-*F*_1,166_ = 69.8; *P* < 0.0001), despite higher concentrations of PAHs and most metals tested in EGSE/CL1 than in EGSE/CL2 (Table 2), indicate lower sensitivity in stage CV copepodites.

**Table 2:** Composition of PAHs and Metals in Undiluted Exhaust Gas Scrubber Effluents (EGSEs) Compared to Intake Seawater Sampled Aboard the Ship Equipped with the First Closed-Loop Scrubber (CL1)^a^

compound	unit	EGSE/CL1	EGSE/CL2	EGSE/OL	seawater
pyrene	ng L^–1^	540	1470	63	4.3
fluoranthene	ng L^–1^	220	1490	222	<1.0
fluorene	ng L^–1^	3200	1380	815	<1.0
acenaphthene	ng L^–1^	2100	454	113	<1.0
acenaphthylene	ng L^–1^	360	37	18	<1.0
anthracene	ng L^–1^	400	<132	<24	<1.0
chrysene	ng L^–1^	330	278	39	<1.0
dibenzothiophene	ng L^–1^	1500			<5.0
methyl-dibenzothiophene	ng L^–1^	8000			<5.0
2/3-methyl-dibenzothiophene	ng L^–1^	3600			<5.0
4-methyl-dibenzothiophene	ng L^–1^	3100			<5.0
dimethyl-dibenzothiophene	ng L^–1^	5900			<5.0
trimethyl-dibenzothiophene	ng L^–1^	5900			<5.0
phenanthrene	ng L^–1^	10,000	5690	2170	<1.0
methyl-phenanthrene	ng L^–1^	25,000			<5.0
dimethyl-phenanthrene	ng L^–1^	22,000			<5.0
trimethyl-phenanthrene	ng L^–1^	1900			<5.0
naphthalene	ng L^–1^	4400	4790	7510	<5.0
dimethyl-naphthalene	ng L^–1^	30,000			<5.0
trimethyl-naphthalene	ng L^–1^	31,000			<5.0
benzo(*a*)anthracene	ng L^–1^	210	231	14	<1.0
benzo(*a*)pyrene	ng L^–1^	<100	14	<10	<5.0
benzo(*b*)fluoranthene	ng L^–1^	100	108	17	<1.0
benzo(ghi)pyrene	ng L^–1^	<100	31	<10	<5.0
benzo(k)fluoranthene	ng L^–1^	70	23	<10	<1.0
dibenzo(ah)anthracene	ng L^–1^	<100	12	<10	<5.0
indeno(*cd*)pyrene	ng L^–1^	<100	11	<10	<5.0
toluene	ng L^–1^	<400			<400
xylene	ng L^–1^	950			<400
1,4-xylene	ng L^–1^	550			<400
1,2-xylene	ng L^–1^	400			<400
benzene	ng L^–1^	1400			<400
hexochlorobenzene	ng L^–1^	<100			<3.0
ethyl-benzene	ng L^–1^	<400			<400
sum 16 US EPA PAH*	ng L^–1^	21,930	16,019	10,981	
total hydrocarbon	μg L^–1^	7106	1960	388	
Al	μg L^–1^	8300	1100	180	1.9
As	μg L^–1^	20	9.8	2.4	39
Cd	μg L^–1^	<0.2	<0.5	<0.5	0.05
Cr	μg L^–1^	9	22	31	<1.2
Cu	μg L^–1^	150	32	14	17
Hg	μg L^–1^	5.2	1.4	6.5	0.84
Ni	μg L^–1^	830	4400	32	0.61
Pb	μg L^–1^	<6	0.16	0.63	0.098
V	μg L^–1^	9800	13,000	84	3.74
Zn	μg L^–1^	<70	46	82	6.2
S	mg L^–1^	19,000	22,000	1200	1100
NO_2_–N	mg L^–1^	49	<0.4	<0.4	<30
NO_3_–N	mg L^–1^	<1	18	0.18	31
pH		7.6	6.9	3.4	7.9
turbidity	NTU	9.3	12.9	2.5	12.9

a Turbidity is expressed as the nephelometric turbidity unit (NTU). * Values below the limit of detection are not included.

### Effects on Metabolism

**Figure 3:**
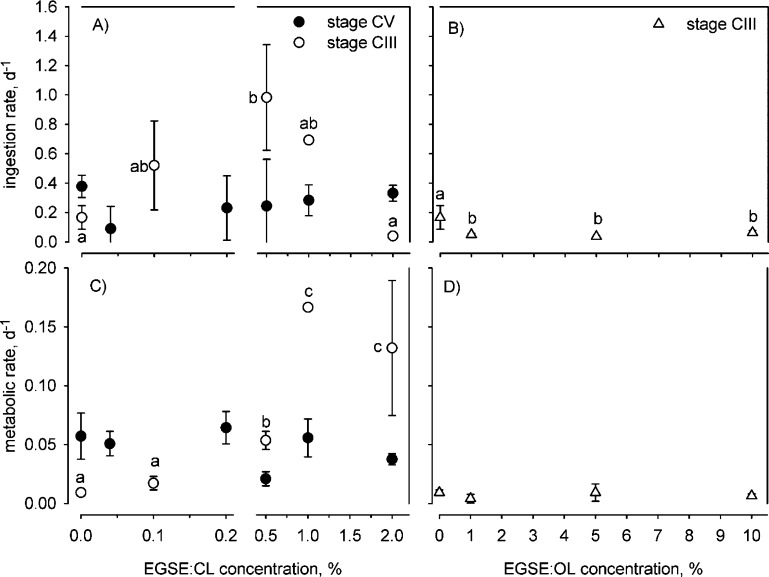
Ingestion rates and metabolic rates of *Calanus helgolandicus* exposed to EGSE. (A) Ingestion rates of stage CV and CIII copepodites exposed to EGSE/CL1 and EGSE/CL2, respectively. (B) Ingestion rates of stage CIII copepodites exposed to EGSE/OL, (C) metabolic rates of stage CV and CIII copepodites exposed to EGSE/CL1 and EGSE/CL2, respectively, and (D) metabolic rates of stage CIII copepodites exposed to EGSE/OL. Lowercase italicized letters indicate statistically equal groups. Letters are absent when the result of the overall statistical tests was nonsignificant.

Besides increased mortality, we also observed sublethal metabolic effects. In stage CIII copepodites exposed to EGSE/CL2, ingestion rates increased sixfold from 0% EGSE to 0.5% and 1% EGSE and then decreased again to 0% EGSE levels at 2% EGSE (1-factor PERMANOVA Pseudo-*F*_4,12_ = 6.10, *P*_MC_ = 0.014, pairwise tests: *P*_MC_ > 0.05; Figure 3A), a reaction also observed in the much smaller copepod species *Oithona davisae* exposed to naphthalene and dimethyl-naphthalene.^33^ Concurrently, metabolic rates increased sixfold from the control treatment to the 2% closed-loop EGSE concentration (1-factor PERMANOVA: Pseudo-*F*_4,14_ = 11.09, *P*_MC_ = 0.0014; Figure 3C). Such a behavior may be an effect of metabolic hormesis, an evolutionary mechanism to counter suboptimal environments.^34,35^ By increasing energy intake, the copepods may have been compensating for the physiological stress imposed by EGSE exposure, whereas at higher EGSE concentrations, this compensation broke down and rates decrease again. We did not find a similar hormesis effect in stage CIII copepodites exposed to EGSE/OL. Here, ingestion rates decreased slightly from 1% and upward compared to the control (1-factor PERMANOVA: Pseudo-*F*_3,11_ = 4.94, *P*_MC_ = 0.030, pairwise tests: *P*_MC_ > 0.05; Figure 3B), whereas their metabolic rates remained unaffected (1-factor PERMANOVA: Pseudo-*F*_3,9_ = 1.10, *P*_MC_ = 0.43; Figure 3D). Also taking into account the lower mortality inflicted by EGSE/OL than by EGSE/CL, this difference probably reflects a lower general toxicity of EGSE/OL. We did not find any significant effects in stage CV copepodites on either the ingestion rate (1-factor PERMANOVA: Pseudo-*F*_6,23_ = 0.911, *P*_MC_ = 0.50; Figure 3A) or metabolic rate (1-factor PERMANOVA pairwise tests: *P*_MC_ > 0.05; Figure 3C), again indicating lower effects in this life stage.

### Population Level Consequences

In nature, mortality rates of late *Calanus* copepodites are in the order of 0.1 d^–1^ and any increased mortality due to EGSE exposure should be superimposed on those.^36,37^ Because we tested effects on a natural population of *C. helgolandicus*, rather than on cultured copepods adapted through generations to only one particular laboratory environment, we are able to infer directly on expected population effects. Our results clearly show that even low concentrations of closed-loop EGSE may double or triple the overall mortality rates of younger copepodite stages in an exposed population. All in all, increased mortality, slowed stage development, and metabolic stress affected stage CIII copepodites to the extent that only 36 and 3% copepodites reached stage CIV during exposure to 0.1 and 0.5% EGSE/CL2, respectively. For EGSE/OL, the numbers were 44 and 4% for concentrations of 5 and 10% EGSE, respectively. This should be compared to the nonexposed copepods in the control treatment where 86% reached the CIV stage during the incubation period.

The negative effects of EGSE release will permeate large parts of the pelagic environment. In the present study, the EGSE discharge rate was ∼10 m^3^ h^–1^ (0.2 m^3^ MW h^–1^ engine power) from the two closed-loop scrubbers and around 35 times as high, ∼350 m^2^ h^–1^ (45 m^3^ MW h^–1^ engine power), from the open-loop scrubber. The lowest observed concentration of EGSE causing a statistically significant effect was only 25 times lower in EGSE/CL1 (0.04%) and 10 times lower in EGSE/CL2 (0.1%) than in EGSE/OL (1%). Thus, the toxic effects of EGSE exposure may be higher from vessels with closed-loop systems operating at nominal engine loads (70–75%), and further studies are needed to fully understand the usefulness of installing closed-loop systems. Decisions must include a proper analysis of the dilution and mixing of both types of EGSE into the water column as the volume release differs tremendously. Although released EGSE will be diluted in the sea, routes frequently trafficked by vessels with scrubbers will constitute regions with elevated EGSE concentrations.^38,39^ Specifically, we envision pelagic “curtains” of intensified EGSE exposure containing high numbers of recently dead copepods and other equally sensitive zooplankters along intensely trafficked shipping lanes. However, EGSE pollution may extend much further than this. Models employing maximum installation scenarios in which all ships with sufficient economic incentive have installed scrubbers show maximum environmental open-loop EGSE concentrations of up to 0.2% in German waters.^39^ We found increased mortality rates already at 1% EGSE/OL, and contamination at these levels may pose a real challenge for pelagic organisms. Moreover, lethargy was significantly increased in stage CIII copepodites already during the first three days of exposure to 2% EGSE/CL2 (2-factor PERMANOVA: pairwise comparison among EGSE concentrations: *P* > 0.05; Figure S3A). Later, lethargy decreased significantly, but this was due to death of these lethargic copepods and not because lethargy among survivors decreased (2-factor PERMANOVA: pairwise comparison among days: *P* > 0.05). There was no increased lethargy in copepodites exposed to EGSE/OL (Figure S3B) (we did not study lethargy in stage CV copepodites). Accordingly, PAHs have been shown to induce narcosis in marine copepods.^40^ Along with recently dead copepods, lethargic copepods constitute easy prey and will, in the high predation environment that is the pelagic, certainly be eaten quickly. Easy prey attracts motile predators, resulting in accumulation of the contaminants in planktivorous predators from a larger area. Trophic transfer and biomagnification constitute serious vectors of transport of toxic metals and organic pollutants along pelagic food webs.^41,42^ The envisioned curtains may also form lethal barriers for invertebrate larvae (pelagic or benthic), thereby constraining progeny dispersal.

### Possible Chemical Origins of Effects

In general, EGSEs vary widely in PAH and metal concentrations. Comparing to a list published by Teuchies and colleagues, our closed-loop EGSEs seem to contain PAHs at concentrations several times higher than the average ship, whereas heavy metal concentrations are slightly lower (except for Hg).^43^ In our open-loop EGSE, PAH concentrations are close to the average, whereas metal concentrations seems lower than average, except Hg which was five times higher than average.^43^

Several constituents of EGSE are potentially toxic to pelagic copepods. Acidity is regulated in EGSE/CL1 and EGSE/CL2 (but not in EGSE/OL) before release, and although average pH_T_ was significantly different among EGSE concentrations for all three EGSEs (PERMANOVA: *P* < 0.0001; Table S2), the dilution of EGSE in the copepod incubations only marginally lowered the seawater pH_T_ so that it remained above 8.0 at the concentrations where significant effects first appeared. *Calanus* copepodites have shown no physiological reaction to pH changes down to 8.0.^24,44^ A_T_ showed significant differences among concentrations (PERMANOVA: *P* ≤ 0.0052; Table S2), but variations were small. Most conspicuously, in the EGSE/OL 40% concentration, *A*_T_ was *ca.* half of that in the rest of the EGSE/OL concentrations.

S concentrations were *ca.* 20 times higher in the closed-loop EGSEs than in the intake seawater but similar to the intake seawater in EGSE/OL (Table 2). Exhaust sulfur is released primarily as SO_2_^–^,^45^ but in seawater, this SO_2_^–^ is rapidly hydrolyzed to SO_3_^2–^, which in turn is almost completely oxidized to SO_4_^2–^ within 24 h.^46−48^ The concentration of S was the highest in EGSE/CL1: 19 g L^–1^. Measurements at the seawater inflow to the scrubber system showed a S concentration of 1.1 g L^–1^, consistent with typical seawater concentrations of SO_4_^2–^, so the addition of closed-loop EGSE at concentrations below 1.1/19 ≈ 6% did not increase sulfur concentrations in the treatment batch water and the toxic action of EGSE is to be found among other compounds.^49^

Polycyclic aromatic hydrocarbons (PAHs) are generally considered the most toxic hydrocarbons in any oil-derived mixtures.^50,51^ They bioaccumulate in copepods and have been shown to induce lowered survival and egg production.^52−54^ We found high concentrations of almost all analyzed PAHs tested in the EGSEs (Table 2). Nonalkylated PAHs were dominated by the two- and three-ring compounds naphthalene, phenanthrene, and fluorene (Table 2). In many studies, analyses of environmental PAHs are limited to only those included in the 16 U.S. EPA standard PAHs.^55^ In our study, none of these occurred in concentrations that according to previously reported toxic effect levels could explain the effects observed. For instance, in female *A. tonsa*, phenanthrene, fluoranthene, and pyrene have shown LC_50_ values above 500 nM (∼100 μg L^–1^) after 48 h of exposure.^53^ We found LC_50_ values of the closed-loop EGSE at *ca.* 3% concentration in stage CV copepodites and *ca.* 1% in stage CIII copepodites, which correspond to measured concentrations more than 2 orders of magnitude lower than these three PAHs. Moreover, EGSE naphthalene concentrations were 4.40, 4.79, and 7.5 μg L^–1^ in undiluted EGSE/CL1, EGSE/CL2, and EGSE/OL, respectively. In comparison, tests of naphthalene toxicity in *O. davisae* showed an LC_50_ for nauplii as high as 4.4 mg L^–1^ and no mortality in adults at concentrations of up to 10 mg L^–1 33^. Besides the 16 standard PAHs, there are hundreds of other PAHs and alkylated, oxygen-, sulfur-, or nitrogen-substituted polycyclic aromatic compounds of which an unknown quantity may be present in the EGSEs. Limiting the analysis to the 16 US EPA PAHs will therefore seriously underestimate the true exposure. When testing 59 nonalkylated and alkylated PAHs, it was found that non-US EPA PAHs constituted 69.3–95.1% of the toxic equivalents as based on toxic equivalent factors (TEF) for 24 PAHs.^56^

Of the alkylated PAHs analyzed in EGSE/CL1, mono-, di-, and trimethylated isomers of naphthalene, phenanthrene, and dibenzothiophene exceeded the concentrations of the parent compounds 5–18 times (Table 2). Alkylated PAHs bioaccumulate at higher rates than their parent compounds due to their higher lipophilicity^57,58^ and observed biological effects have been attributed to alkylated rather than parent PAHs in organisms from bacteria to mussels, fish, and sea otters.^59−63^ Due to the high contents of alkylated PAHs observed where they were measured, in EGSE/CL1, we therefore hold these compounds as likely candidates for the observed toxic effects. On the other hand, in the sole previously published study on copepod toxic effects from alkylated PAHs, Saiz *et al.*(33) found LC_50_ values of dimethyl-naphthalene at 771 μg L^–1^ in nauplii and 1346 μg L^–1^ in adult *O. davisae*, whereas the closed-loop EGSE concentration of dimethyl-naphthalene was much lower at 30 μg L^–1^ (Table 2).

The concentrations of dioxins/furans and hexachlorobenzene were below the detection limits, which for the dioxin/furan congeners varied between 0.91 and 3.6 pg L^–1^ and for hexachlorobenzene was 100 ng L^–1^. Other monoaromatic compounds were found in concentrations between 400 and 950 ng L^–1^, except for toluene and ethylbenzene which were below the detection limit (Table 2).

Concentrations of Al, Cr, Cu, Hg, Ni, V, and Zn were considerably higher in all three EGSEs than in the intake seawater (Table 2). Hg significantly reduces egg production in *Acartia* spp. at concentrations down to 50 ng L^–1^.^64^ The EGSEs contained Hg at 5.2, 1.4, and 6.5 ng L^–1^ concentrations (EGSE/CL1, EGSE/CL2, and EGSE/OL, respectively), so EGSE Hg did not cause the toxic response we observed. 48 h LC_50_ of Cu has been established at *ca.* 120 μg L^–1^ in female *A. tonsa*,^65^ 24 h LC_50_ of *ca.* 180 μg L^–1^ in *Scutellidium* sp.,^66^ and 96 h LC_50_ of 64 and 88 μg L^–1^ in nauplii and adults of *Tisbe battagliai*, respectively.^67^ These are equivalent to the concentration of Cu in undiluted closed-loop EGSE, whereas we observed increased mortality already at concentrations 3 orders of magnitude lower (0.04 and 0.1%). Similarly, for Ni, the 96 h LC_50_ is 136 μg L^–1^ in the copepod *Pseudodiaptomus marinus,*(68) whereas Ni concentrations were 0.3, 4.4, and 0.3 μg L^–1^ (EGSE/CL1, EGSE/CL2, and EGSE/OL, respectively) at the lowest effect EGSE concentrations. Koski *et al.*(16) observed elevated levels of Cu, Ni, V, and Pb in the EGSE from the tested open-loop scrubber along with the increasing mortality of adult *A. tonsa*. Concurrent with the higher copepod mortality compared to our study, the EGSE/inflow-concentration ratio of V was as high as 257 in Koski *et al.* (2007) but only 22.7 for EGSE/OL in our study. This could indicate that the V content of the EGSE/OL influenced copepod mortality in both studies. Koski *et al.* (2007) also observed EGSE/inflow-concentration ratios of Cr and Zn at 4.9 and 2.0, while we observed ratios of 25.8 and 13.2, respectively. This is despite the higher observed mortality in the Koski *et al.* (2017) study. Assuming similar sensitivities toward EGSE of *A. tonsa* and *C. helgolandicus*, it follows that the mortality we observed in *C. helgolandicus* was not caused by Cr or Zn. However, it should be noted that the toxicity of metals depends very much on their speciation. We analyzed only the total concentration of the metals in the EGSEs and not their species.

The NO_2_^–^ concentration was 49 mgN L^–1^ in EGSE/CL1. Little is known about the toxic effects of NO_2_^–^ in copepods, but the prawn *Penaeus monodon* has shown 24 h LC_50_ values of 5.00 mgN L^–1^ in nauplii and 13.20 mgN L^–1^ in zoea larvae.^69^ Calculated NO_2_^–^concentration in the 0.04% EGSE/CL1 treatment was *ca.* 2 μg N L^–1^, more than 3 orders of magnitude lower and comparable to the concentration in coastal sea water.

### Witch’s Cauldrons

In summary, none of the contaminants we tested for and found prior established copepod toxicity levels for (except perhaps V) occurred in concentrations that alone could explain the toxicity of the tested EGSEs. The measured toxicity may arise from compounds not a or be caused by synergistic effects among several contaminants. For instance, both EGSE/CL2 and EGSE/OL contained high concentrations of Zn, Cr, and Ni and previous studies show synergistic effects between Zn and Ni and between Zn and Cr in copepods,^70^ whereas other studies showed additive effects.^71^ Also, combined effects among PAHs and between metals and PAHs have been observed previously.^40,72^ Moreover, while the PAH content of the fuel oil itself and the products normally formed during its combustion may be known,^73^ the result of mixing compounds such as metals, NO_*X*_, SO_*X*_, and organics in the scrubber where both temperature and pH vary greatly is largely unknown. The homologue profiles of the alkylated PAHs did not form the descending trend expected for pyrogenic PAHs, with the highest concentrations for the parent compound and consecutively decreasing concentrations with increasing number of alkylation groups (Table 2).^73,74^ It is likely that scrubbers act as witch’s cauldrons where various chemicals mix, reactions occur, and undesired toxic compounds form.

In conclusion, the present study clearly shows that effluents from maritime scrubber systems, whether they originate from open-loop or closed-loop systems, are highly toxic to zooplanktonic organisms. Our results provide a strong environmental rationale to avoid the use of maritime scrubbers. While the intentions of the IMO may have been to find an environmentally sound solution to the sulfur problem, scrubbers effectively just function to move pollution from the atmosphere to the sea, thereby creating a suite of new unwanted environmental problems.^43^ Moreover, allowing the use of scrubbers also economically incentivizes increased use of residual heavy fuel oils high in PAHs and heavy metals, with an accompanying increased environmental toll, instead of development of fuels with less environmental impact.

## Supporting Information Available

Methods; incubations; exposure concentration, copepodite stage, exposure time, and sampling periodicity of the three experiments; chemical analysis of EGSE; pH and total alkalinity of treatment water; results; pH and total alkalinity; cumulative mortality; cumulative molting; and cumulative lethargy (PDF)
Raw data on mortality rates, molting rates, ingestion rates, and metabolic rates (XLSX)
