# Supplementary material for: Severe Toxic Effects on Pelagic Copepods from Maritime Exhaust Gas Scrubber Effluents
Source: Environ Sci Technol. 2021 Apr 20;55(9):5826–35. doi: 10.1021/acs.est.0c07805 (PMC8154367; doi:10.1021/acs.est.0c07805)
Supplement: Supplementary file 1 — es0c07805_si_001.pdf [file es0c07805_si_001.pdf]

Supporting information for:

Severe toxic effects on pelagic copepods from maritime exhaust gas scrubber effluents

Peter Thor[1*+], Maria E. Granberg[2], Hulda Winnes[2], Kerstin Magnusson[2]

[1] Norwegian Polar Institute, Fram Centre, 9296 Tromsø, Norway*

[2] IVL Swedish Environmental Research Institute, Kristineberg Marine Research Station, Kristineberg 566, 451 78 Fiskebäckskil, Sweden

* Present address: Swedish University of Agricultural Sciences, Department of Aquatic Resources, Institute of Marine Research, Turistgatan 5, 45330 Lysekil, Sweden

[+] corresponding author: peter.thor@slu.se

Number of pages: 6

Number of tables: 2

Number of figure: 3

Methods

Incubations

Table S1. Exposure concentration, copepodite stage, exposure time, and sampling periodicity of the three experiments.

	EGSE:CL1	EGSE:CL2	EGSE:OL
**Control**	0 %	0 %	0 %
**Conc. 1**	0.04 %	0.1 %	1 %
**Conc. 2**	0.2 %	0.5 %	5 %
**Conc. 3**	0.5 %	1 %	10 %
**Conc. 4**	1 %	2 %	40 %
**Conc. 5**	2 %	5 %	-
**Conc. 6**	5 %	-	-
**Copepodite stage**	CV	CIII	CIII
**Exposure time (d)**	7	8	14
**Sampling / water change**	Daily	Daily	Every second day

Chemical analysis of EGSEs

Samples were collected from all three EGSEs for analysis of total hydrocarbons (including 16 U.S. EPA PAHs, hexachlorobenzene, dioxins and furans) (acetone washed dark glass bottles with Teflon lid), metals, sulphur, NO_X, turbidity, pH and alkalinity (acid washed plastic bottles). In port, these samples were packed and shipped overnight to the respective analysis laboratories.

All three EGSEs were analysed for total hydrocarbon content and the 16 U.S. EPA PAHs, naphthalene, acenaphthylene, acenaphthene, fluorene, phenanthrene, anthracene, fluoranthene, pyrene, benzo(a)anthracene, chrysene, benzo(b)fluoranthene, benzo(k)fluoranthene, benzo(a)pyrene, dibenzo(ah)anthracene, benzo(ghi)perylene and indeno(1,2,3-cd) pyrene (Table 1). In addition, EGSE:CL1 was analysed for the monoaromatic compounds, the PAH dibenzothiophene and alkylated isomers of the PAHs naphthalene (di- and trimethylnaphthalene), phenanthrene (mono- di- and trimethylphenanthrene) and dibenzothiophene (monomethylated isomers, methyldibenzothiophene, 2/3 methyldibenzothiophene and 4- methyldibenzothiophene, and di- and trimethylated dibenzothiophene) and polychlorinated dibenzo-p-dioxins (PCDDs), polychlorinated dibenzofurans (PCDFs). All samples were analysed for the metals aluminium (Al), arsenic (As), cadmium (Cd), chromium (Cr), copper (Cu), nickel (Ni), lead (Pb), zinc (Zn), vanadium (V) and mercury (Hg), and also for sulphur (S), nitrite (NO_2-N), nitrate (NO_3- N), conductivity, pH, alkalinity and turbidity (Table 2). Samples were also collected from the seawater intake on board one of the ships equipped with a closed loop scrubber system (CL1) and analysed for the same parameters as EGSE:CL1. Analysis of total hydrocarbon fractions, PAHs and alkylated PAHs in EGSE:CL1 was completed within 72 hours, and analyses of other organic contaminants (monoaromatics, PCBs and dioxins) were completed within a week. Total hydrocarbon fractions

and PAHs in EGSE:CL2 and EGSE:OL were analysed within two weeks after sampling. Turbidity was analysed within 48 hours after sampling and metals and S, NOx, pH, alkalinity and turbidity within one week after sampling.

Total hydrocarbons (THC) and PAH in EGSE:CL1 and in EGSE:CL2 and EGSE:OL were analysed at two separate laboratories, Water Management Laboratories, Povodí Labe, Státní Podnik, Czech Republic and ALS Scandinavia AB. For EGSE:CL1, THC was analysed using a method published by New Jersey Department US EPA (NJDEP EPH 10/08, Analysis of Extractable Petroleum Hydrocarbon Compounds in Aqueous and Soil/Sediment/Sludge Matrices), where acidified samples where extracted with dichloromethane and analysed with gas chromatography with flame ionization Detector (GC-FID). PAH 16, alkylated PAHs and HCB in EGSE:CL1 were determined by gas chromatography-mass spectrometry (GC-MS) according to analytical standard method CSN ISO 28540:2012 with a few validated changes. Determination of PCDD/Fs in EGSE:CL1 was done with the isotope dilution method using HRGC-HRMS. The procedure fulfils the conditions and requirements of the EPA standard 1613. For EGSE:CL2 and EGSE:OL, THC was analysed with GC-FID according to CSN EN ISO 9377-2 and PAHs were determined by GC-MS according to a method based on US EPA 8270 and CSN EN ISO 6468 with a few validated changes.

For the analysis of metals and sulphur, samples were treated with HNO_3 and analysed with Inductively Coupled Plasma-Quadropole Mass Spectrometry (ICP-QMS) with interference elimination by collision cell with helium. Nitrate and nitrite were analyzed on an anion chromatograph (Thermo Scientific, Dionex). After the samples were passed through an anion exchange column separating the ions, the conductivity of the anions was determined with a conductivity detector. Carbonate alkalinity was determined by titrating the samples under nitrogen gas flow with hydrochloric acid to pH 5.4 according to a modification of SS-EN ISO 9963-2. The pH and conductivity were determined with a pH electrode and a conductivity electrode. Turbidity was determined using a nephelometer detection according to CSN EN ISO 7027-1 and expressed in nephelometric turbidity units (NTU).

Results

Table S2. Total scale pH (pH_T) and total alkalinity (A_T) (means ± standard deviations) during copepod incubations.

**Stage CV, EGSE:CL1**			**Stage CIII, EGSE:CL2**			**Stage CIII, EGSE:OL**		
Conc. %	pH_T	A_T	Conc. %	pH_T	A_T	Conc. %	pH_T	A_T
0	8.060 ± 0.022	2496 ± 5	0	8.073 ± 0.030	2 361 ± 6	0	8.073 ± 0.030	2 361 ± 6
0.04	8.061 ± 0.024	2417 ± 9	0.1	8.052 ± 0.038	2 348 ± 13	1	8.031 ± 0.053	2 346 ± 11
0.2	8.027 ± 0.028	2515 ± 28	0.5	8.000 ± 0.030	2 366 ± 24	5	7.915 ± 0.057	2 177 ± 3
0.5	8.012 ± 0.020	2497 ± 3	1	7.890 ± 0.029	2 294 ± 1	10	7.748 ± 0.129	2 017 ± 2
1	7.945 ± 0.044	2498 ± 6	2	7.720 ± 0.035	2 191 ± 16	40	7.163 ± 0.257	1 011 ± 41
2	7.940 ± 0.020	2493 ± 2	5	7.454 ± 0.008	2 241 ± 23			
5	7.936 ± 0.003	-						

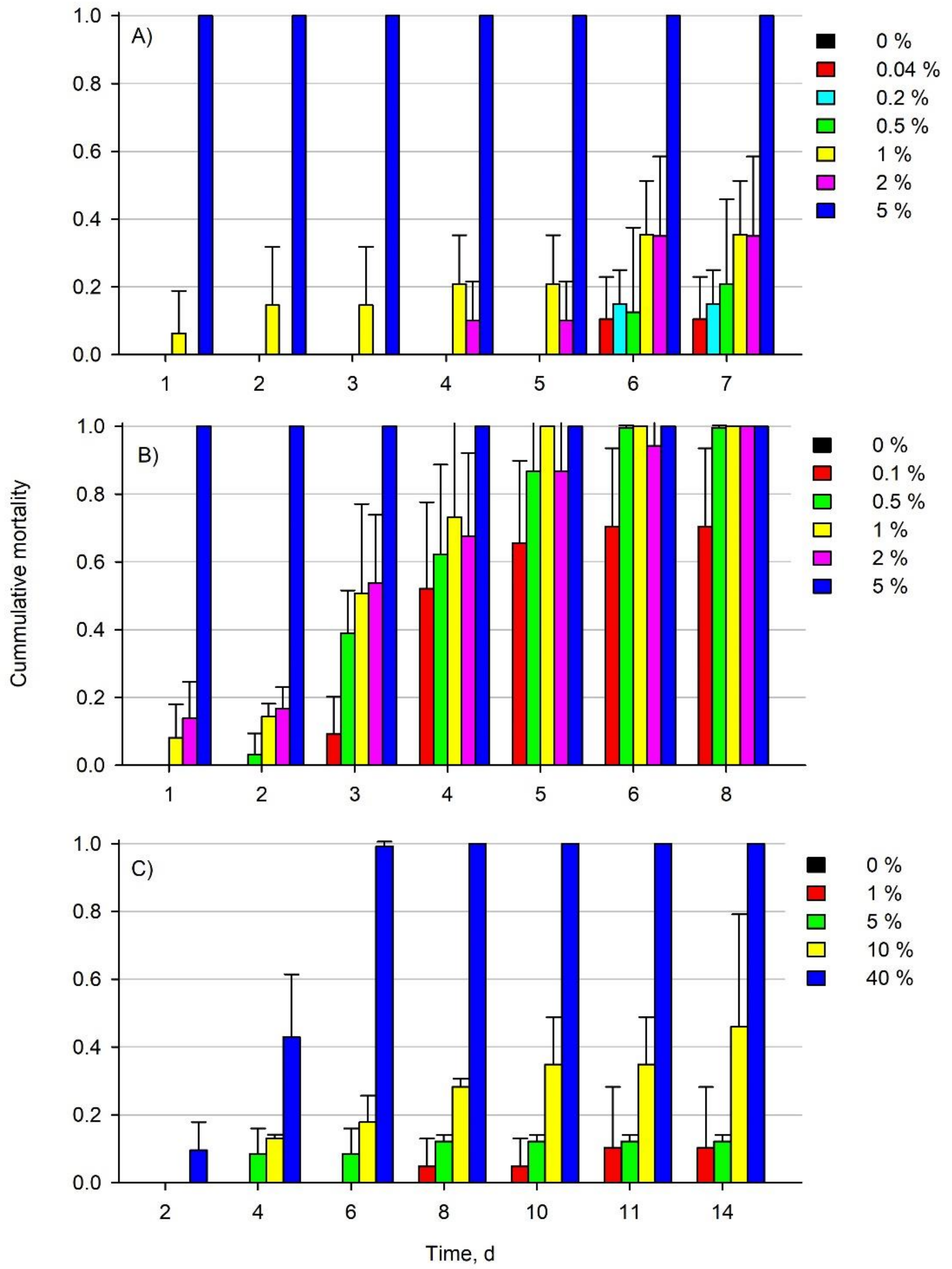

Figure S1. Cumulative fraction dead *Calanus helgolandicus* copepodites. A) Stage CV copepodites exposed to 7 concentrations of EGSE:CL1, B) Stage CIII copepodites exposed to 6 concentrations of EGSE:CL2, C) Stage CIII copepodites exposed to 5 concentrations of ESGE:OL. Means ± standard deviations.

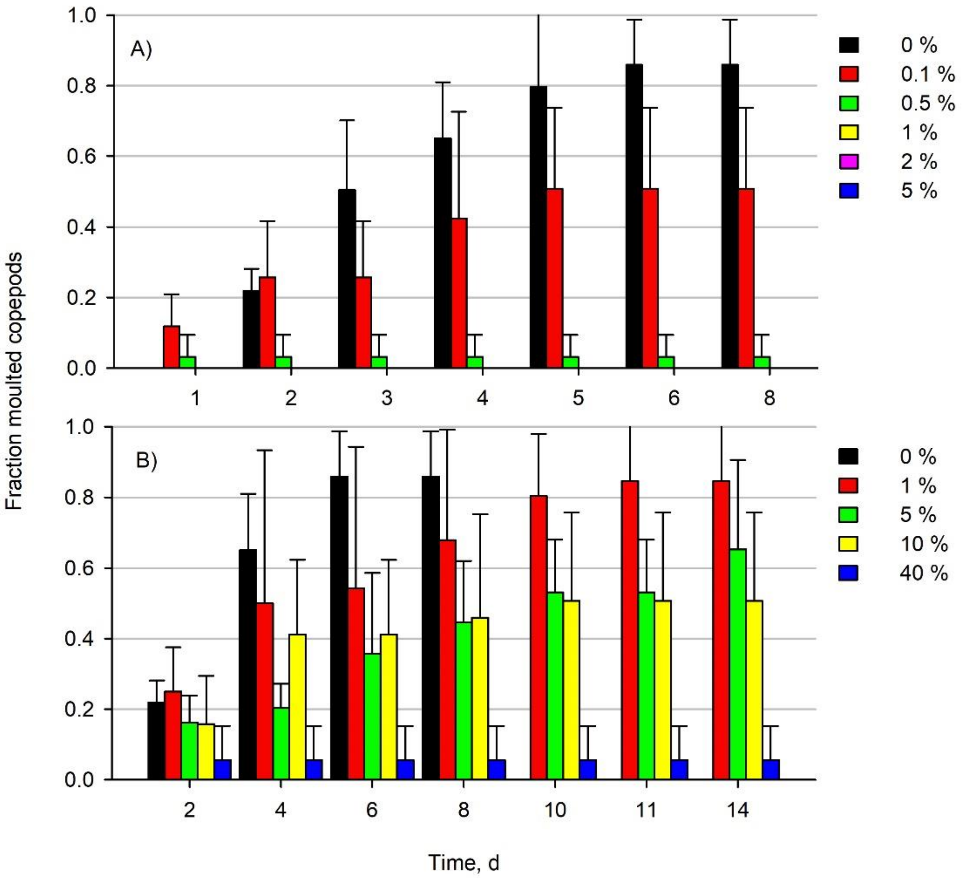

Figure S2. Cumulative fraction moulted *Calanus helgolandicus* stage CIII copepodites . A) Stage CIII copepodites exposed to 6 concentrations of EGSE:CL2, and B) *Calanus helgolandicus* CIII exposed to 5 concentrations of ESGE:OL. Means ± standard deviations.

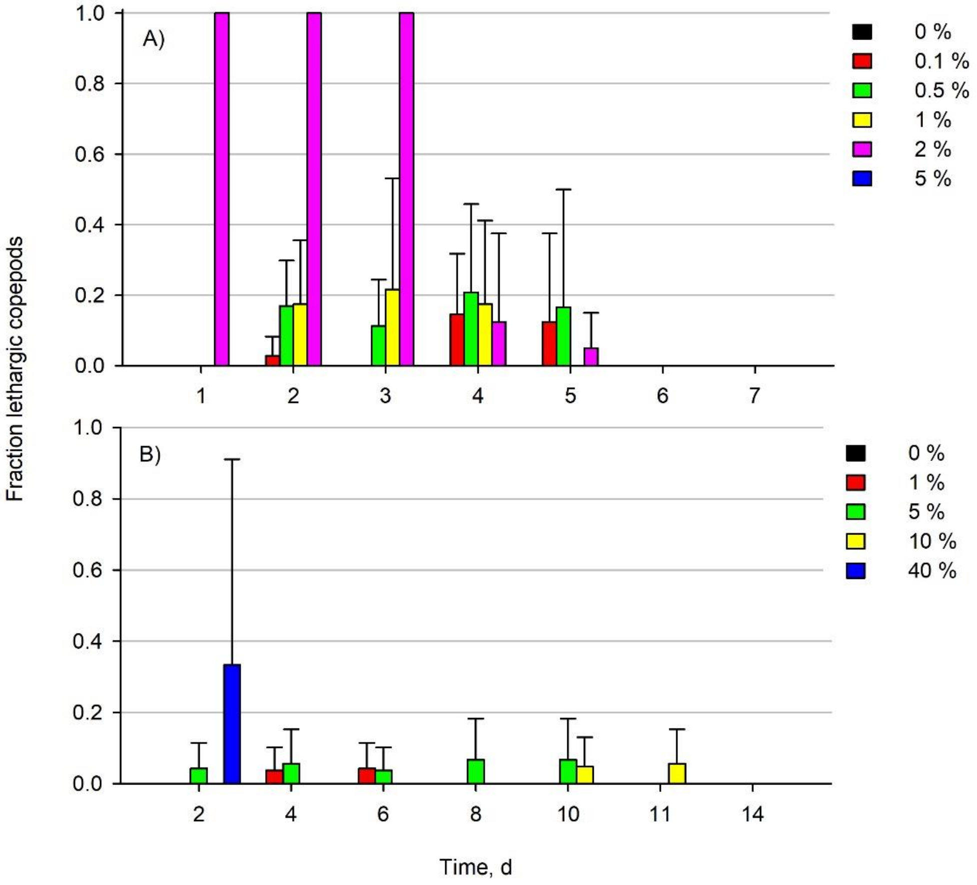

Figure S3. Cumulative fraction lethargic *Calanus helgolandicus* copepodites relative to the number of live copepods. A) Stage CIII copepodites exposed to 6 concentrations of EGSE:CL2, and B) Stage CIII copepodites exposed to 5 concentrations of EGSE:OL. Means ± standard deviations.
